# The psychometric properties of Beck Depression Inventory for adolescent depression in a primary-care paediatric setting in India

**DOI:** 10.1186/1753-2000-1-8

**Published:** 2007-08-09

**Authors:** Mona Basker, Prabhakar D Moses, Sushila Russell, Paul Swamidhas Sudhakar Russell

**Affiliations:** 1 Department of Child Health, Christian Medical College, Vellore 632 002, India; 2 Child and Adolescent Psychiatry Unit, Department of Psychiatry, Christian Medical College, Vellore 632 002, India

## Abstract

**Background:**

There is increasing interest in identifying adolescents with depression in primary care settings by paediatricians in India. This article studied the diagnostic accuracy, reliability and validity of Beck Depression Inventory (BDI) while used by paediatricians in a primary care setting in India.

**Methods:**

181 adolescents attending 3 schools were administered a back translated Tamil version of BDI by a paediatrician to evaluate its psychometric properties along with Children's Depression Rating Scale (CDRS-R) for convergent validity. Clinical diagnosis of depressive disorders, for reference standard, was based on ICD-10 interview by an independent psychiatrist who also administered the Impact of Event Scale (IES) for divergent validity. Appropriate analyses for validity and diagnostic accuracy both at the item and scale levels were conducted.

**Results:**

A cut-off score of ≥ 5 (Sn = 90.9%, Sp = 17.6 %) for screening and cut-off score of ≥ 22 (Sn = 27.3%, Sp = 90%) for diagnostic utility is suggested. The 4 week test – retest reliability was good (r = 0.82). In addition to the adequate face and content validity, BDI has very good internal consistency (α = 0.96), high convergent validity with CDRS-R (r = 0.72; P = 0.001), and high discriminant validity with IES (r = 0.26; P = 0.23). There was a moderate concordance rate with the reference standard (54.5%) in identifying depression among the adolescents. Factor analysis replicated the 2-factor structure explaining 30.5 % of variance.

**Conclusion:**

The BDI proved to be a psychometrically sound measure for use by paediatricians in a primary care setting in India. The possibility of screening for depressive disorders through the use of BDI may be helpful in identifying probable cases of the disorder among adolescents.

## Background

Depressive disorders are identified by the World Health Organization as *priority mental health disorder *of adolescence because of its high prevalence, recurrence, ability to cause significant complications and impairment [1]. Across the globe, the lifetime prevalence for major depression in adolescence is 15% to 20% [2] with a recurrence rate of 60–70% [3] often resulting in suicide, school dropout, pregnancy, substance abuse, progressing in to adult depression [4,5], functional disability and significant impairment [6].

In India, paediatricians are often the first step in the pathway to mental health and sometimes the only contact for an adolescent with a health professional for a myriad of mental health problems including depression and thus paediatrician's role in identifying depression in these adolescents becomes indispensable. The prevalence of depression among adolescents among primary-care paediatric care settings in India is 11.2% [7] and recognizing adolescent depression becomes a responsibility of paediatricians [8]. However, up to 50% of depressed adolescents are not diagnosed in primary-care settings [9]. Although several depression screening instruments are available, their psychometric properties in a primary-care paediatric setting in the Indian context has note been studied. Beck Depression Inventory (BDI) has excellent psychometric properties across clinical and non-clinical populations in other countries [10]. BDI has also been extensively validated among the adolescent population elsewhere [11]. Therefore this study was conducted to document the psychometric properties of BDI in a primary-care setting in India while being used by paediatricians.

## Methods

### Setting and participants

Participants were recruited from three schools at Vellore that represent the higher (Private ICSC board school), middle (Private matriculation board school), lower socio-economic (Public state board school) backgrounds and they represent the literate young adolescent population in India. All adolescents were included in the study if they were in the 11 grade (to avoid the symptoms of depression due to educational stress of appearing for board examination in the 10^th ^as well as the 12^th ^grades), and able to read and write English atleast at sixth grade level. Knowledge of English was required to effectively administer measures other than BDI that were required in the validation procedures and were available only in English (e.g. CDRS-R). Adolescents who satisfied the selection criteria (N = 181) were interviewed and assessed using the following clinical diagnostic criteria and psychometrically sound measures respectively.

### Measures

*Beck Depression Inventory (BDI) *[12] is a 21 item, self rated inventory with each item rated with a set of four possible answer choices of increasing intensity. When the test is scored, a value of 0 to 3 is assigned for each answer and then the total score is compared to a key to determine the depression's severity. It can be administered for adolescents above 14 years as the reading level of the measure is only at sixth grade level and can be completed in about 10 minutes. The reliability and validity of BDI has been demonstrated with adolescents in other countries [13]. BDI was chosen over BDI-II because it's available free of cost for the primary-care users in India, a low income country. The Tamil version of the BDI after the back translation procedures was the measure for validation in this study.

*Children's Depression Rating Scale-Revised (CDRS-R) *[14,15] is a clinician rated instrument that covers 17 symptom areas of depression and used to diagnose depression and can be repeated to measure response to treatments. CDRS-R total scores range from 17 to 113 and Fourteen of the 17 items are rated on a scale from 1 to 7, with an item score of 3 suggestive of mild, 4 or 5 moderate, and 6 or 7 severe symptoms. The other 3 items are rated on a scale from 1 to 5. Both children and their parents provide input into the first 14 items of the scale. A child's nonverbal behaviour is rated by the observer for items 15 through 17. A CDRS-R ≥ 40 suggests the presence of depressive disorder. CDRS-R was administered to determine the convergent validity of BDI.

*Impact of Events scale 8 item version (IES-8) *[16,17] has been validated in Tamil for identifying adolescents with Post-Traumatic Stress Disorder (PTSD). The 8-item version of Impact of Event Scale has the intrusive sub-scale as well as avoidance sub-scale with 4 items each. Each item is scored positively, with the levels of endorsement valued at 0, 1, 3, and 5 respectively and a score of 17 or higher was considered a cause for clinical concern [16]. IES-8 was used to determine the discriminant validity of BDI.

*The ICD-10 Classification of Mental and Behavioural Disorders *(Clinical Descriptions and Diagnostic Guidelines) [18] based clinical interview with emphasis on Depressive disorders (F32.0, F32.1, F32.2, F32.3, F32.8, F32.9), Recurrent depressive disorders (F33.0, F33.1, F33.2, F33.3, F33.4, F33.8, F33.9), dysthymia (F34.1), mixed anxiety and depressive disorder (F41.2), adjustment disorders including pathological grief (F43.20, F42.21, F43.22) was used as the reference standard. Because of proven international, standard diagnostic classification utility for general practice and mood disorders research of ICD-10 [19,20] it was used as the reference standard.

### Interview and assessment

Participants were interviewed by three researchers, a consultant paediatrician, followed by a consultant clinical psychologist and finally a consultant psychiatrist on the same or subsequent week at school. The paediatrician administered the self-rated BDI and assessed the adolescents with CDRS-R. The clinical psychologist independently rated the adolescents with CDRS-R to maintain inter-rater reliability of CDRS-R, being used for the convergent validity. The psychiatrist, independently, interviewed the respondents to diagnose the possibility of an ICD-10 category of psychiatric disorder, especially the depressive disorders using a semi-structured clinical interview and administered the Impact of Event Scale as well. The semistructured psychiatric interview, we devised, was conducted using the section on Depressive Disorders (dysthymia and major depression) in the Kiddie-Sads-Present and Lifetime Version (K-SADS-PL) with modifications to cover specific ICD-10 Depressive Disorders criteria [19]. As these three assessments were independently done, the data was also blinded. The data were collected after the parent provided informed consent and verbal assent of the participants well as the teacher. The BDI was reissued after 4 weeks to 20% of the study sample by the paediatrician to measure the reproducibility. The local Institutional Review Board of Christian Medical College reviewed and provided approval for the study.

### Data analysis

As part of the data analysis, preliminary checks of skewness verified that our data were suitable for parametric analysis and the psychometric properties of BDI were analysed at both the item and scale levels. *Sensitivity *and s*pecificity *for various BDI cut-off scores were calculated in order to determine the optimal screening as well as diagnostic threshold with Receiver operating characteristic (ROC) analyses and contingency tables. The *test-retest reliability *of BDI was examined with the intra class correlation. For *internal consistency*, Cronbach's α coefficient was calculated. The concordance rate between BDI threshold score and the ICD-10 based diagnosis was determined with Cohen's Kappa test. To determine the *convergent validity *and *criterion validity *of the BDI as a self-rated measure of depression, the total score of BDI was correlated with the CDRS-R and clinical diagnosis of depression using ICD-10 respectively. The concordance (overlapping cases) of the ICD-10 diagnosis of depression and BDI diagnosis of depression was computed as the quotient of the cases classified as depression by both of the measures applied and the number of cases classified as depression by either of the measure. *Discriminant validity *was calculated by correlating BDI score with IES, as it was hypothesized that CDRS-R diagnosis of depression would be more closely related to the BDI scores than IES scale measuring Post-traumatic Stress Disorder. The *Factor structure *of BDI was demonstrated by principal components analysis with promax rotation. Data was analysed using SPSS software version 12.

## Results

### Sample characteristics

Of the 181 adolescents interviewed, sample full data was available for only 178 participants. The mean (sd) age of the adolescents was 15.6(0.6) with a range of 14 to 17 years. There was a mild over representation of boys (N = 105) than girls (N = 73) in the sample. The mean (sd) BDI score was 13.4(8.3) with a range of 0 to 42 and CDRS-R score was 27.5(8.2) with a range of 17 to 54. Among the participants identified as having a depressive disorder (N = 11), the most prevalent diagnostic group had mild, moderate or severe depression depressive episode with somatic symptoms (N = 5), followed by Brief depressive reaction (N = 3), Mixed anxiety-depression (N = 2) and finally grief (N = 1).

### Diagnostic accuracy

**Figure 1:**
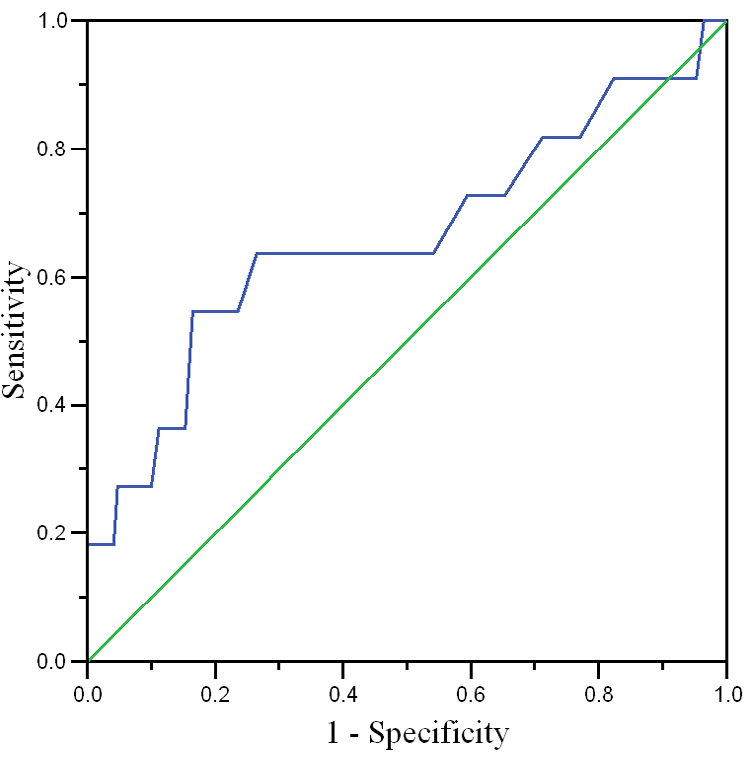
Receiver operating characteristic (ROC) curve for the total BDI score.

For the sensitivity and specificity, differing cut-off points for the BDI were tested. Table 1 summarizes these results. A score of ≥ 5 in BDI achieved sensitivity between 80–100% and therefore was ideal as a screening cut-off score, where as a score of ≥ 22 had a specificity of 80–100% making it appropriate for a diagnostic use to establish ICD-10 diagnosis of Depression. The area under curve (AUC) in the ROC for the BDI was 0.66 with a standard error of 0.1 as noted in Figure 1.

**Table 1:** Specificity and sensitivity of different cut-off scores on the BDI with ICD-10 clinical diagnosis as gold standard.

**Cut-off score**	Sensitivity (%) (95% CI)	Specificity (%) (95% CI)	**Cut-off score**	Sensitivity (%) (95% CI)	Specificity (%) (95% CI)
**≥ 1**	100.0 (71.3–100.0)	3.5 (1.3–7.5)	**≥ 19**	54.5 (23.5–83.1	83.5 (77.1–88.8)
**≥ 2**	90.9 (58.7–98.5)	4.7 (2.1–9.1)	**≥ 20**	36.4 (11.2–69.1	84.7 (78.4–89.8)
**≥ 3**	90.9 (58.7–98.5)	7.1 (3.7–12.0)	**≥ 21**	36.4 (11.2–69.1)	88.8 (83.1–93.1)
**≥ 4**	90.9 (58.7–98.5)	11.2 (6.9–16.9)	**≥ 22**	**27.3 (6.3–60.9)**	**90.0 (84.5–94.1)**
**≥ 5**	**90.9 (58.7–98.5)**	**17.6 (12.2–24.2)**	**≥ 24**	27.3 (6.3–60.9)	90.6 (85.2–94.5)
**≥ 6**	81.8 (48.2–97.2	22.9 (16.9–30.0)	**≥ 25**	27.3 (6.3–60.9)	91.2 (85.9–95.0)
**≥ 7**	81.8 (48.2–97.2	28.8 (22.1–36.3)	**≥ 26**	27.3 (6.3–60.9)	92.9 (88.0–96.3)
**≥ 8**	72.7 (39.1–93.7	34.7 (27.6–42.4)	**≥ 27**	27.3 (6.3–60.9)	94.7 (90.2–97.5)
**≥ 9**	72.7 (39.1–93.7	37.6 (30.3–45.4)	**≥ 28**	27.3 (6.3–60.9)	95.3 (90.9–97.9)
**≥ 10**	72.7 (39.1–93.7)	40.6 (33.1–48.4)	**≥ 29**	18.2 (2.8–51.8)	95.9 (91.7–98.3)
**≥ 11**	63.6 (30.9–88.8)	45.9 (38.2–53.7)	**≥ 30**	18.2 (2.8–51.8)	95.9 (91.7–98.3)
**≥ 12**	63.6 (30.9–88.8)	52.4 (44.6–60.1)	**≥ 32**	18.2 (2.8–51.8)	97.6 (94.1–99.3)
**≥ 13**	63.6 (30.9–88.8)	55.3 (47.5–62.9)	**≥ 33**	18.2 (2.8–51.8)	98.8 (95.8–99.8)
**≥ 14**	63.6 (30.9–88.8)	61.2 (53.4–68.5)	**≥ 34**	18.2 (2.8–51.8	99.4 (96.8–99.9)
**≥ 15**	63.6 (30.9–88.8)	66.5 (58.8–73.5)	**≥ 37**	18.2 (2.8–51.8	100.0 (97.8–100.0)
**≥ 16**	63.6 (30.9–88.8)	70.6 (63.1–77.3)	**≥ 40**	9.1 (1.5–41.3	100.0 (97.8–100.0)
**≥ 17**	63.6 (30.9–88.8)	73.5 (66.2–80.0)	**≥ 42**	0.0 (0.0–28.7)	100.0 (97.8–100.0)
**≥ 18**	54.5 (23.5–83.1)	76.5 (69.4–82.6)			

### Reproducibility

The *test-retest reliability *was studied to assess the reproducibility of BDI and the intra class correlation coefficient was found to be good (r= 0.82). The Cronbach's α coefficient for the whole scale was high (α = 0.96) suggesting that the BDI in this population has satisfactory *internal consistency*.

### Validity

None of the 21 items was assigned a score of 0 by more than half of the adolescents with depression in this study suggesting that the *Content validity *was appropriate to their morbid state. The convergent validity between the BDI and CDRS-R, calculated with Pearson correlation was also high (r = 0.72) and significant at the 0.001 level. There was a moderate *concordance rate *between the BDI cut-off score of ≥ 22 and reference standard of ICD-10 diagnosis (54.5%) in identifying depression among the adolescents. While determining the *criterion validity*, the correlation between the diagnosis of cases based on ICD-10 by the psychiatrist and self-rated BDI score was low but was at 0.05 level of significance (r = 0.15, P = 0.04). Eleven of the 178 adolescents who received psychiatric assessment fulfilled criteria for depressive disorders and thus more than three-quarter of the patients failed to meet the ICD-10 criteria for depressive disorders. Among the 178 participants, the paediatrician with BDI recognized 81.8% of participants as depressed and yet, 45.0% of patients labelled as depressed by the paediatrician based on BDI score were not cases of depression according to ICD-10 criteria. It is interesting to note that a large proportion of adolescents were not found to be suffering from any other specific psychiatric disorder by ICD-10 (N = 170) or BDI (N = 65). Other disorders noted were Specific learning disorder (N = 2), Obsessive compulsive disorder (N = 1), Dhat syndrome (N = 1), Tension head ache (N = 1) and 40% of these disorders were also picked as depression by the BDI. *Divergent validity *calculated by correlating BDI scores to the IES showed non-significant associations (r = 0.23; P = 0.26) demonstrating that the BDI discriminates depression from other psychiatric disorders like PTSD.

To investigate the factor structure of the items in the BDI, we extracted a two factors and factor loadings 0.50 were considered significant. The two factors were *Somatic symptoms *with an eigen value of 4.8 and *Mood-negative cognitions *with an eigen value of 1.5. BDI items 10 (crying), 11 (agitation), 16 (changes in sleep pattern) and 20 (tiredness or fatigue) loaded on to *factor 1 (Somatic symptoms)*, BDI items 3 (past failure), 6 (punishment feelings), 7 (self-dislike) loaded on to *factor 2 (Mood-negative cognitions)*. BDI items 1, 2, 4, 5, 8, 9, 12, 13, 14, 15, 17, 18, 19, 21 cross-loaded in to factor 1 and 2, thus were considered not specific to any domain of depression. Otherwise, all items loaded distinctively and without cross-loadings (Table 2).

**Table 2:** Factor loadings of the 21-item two-factor structure of the BDI ^a, b^

	Factors	
BDI items	Somatic symptoms	Mood-negative symptoms
1. sadness	.48	.19
2. Pessimism	.21	.35
3. Past failure	-.20	**.76**
4. Loss of pleasure	.34	.14
5. Guilty feelings	.27	.17
6. Punishment feelings	.01	**.58**
7. Self dislike	-.06	**.79**
8. Self criticalness	.46	.03
9. Suicidal thoughts and wishes	.27	.35
10. Crying	**.67**	-.21
11. Agitation	**.74**	-.29
12. Loss of interest	.20	.19
13. Indecisiveness	.42	.25
14. Worthlessness	.17	.42
15. Loss of energy	.46	-.01
16. Changes in sleeping pattern	**.51**	-.06
17. Irritability	.44	.22
18. Changes in appetite	.31	.33
19. Concentration difficulty	.48	.02
20. Tiredness or fatigue	**.67**	.06
21. Loss of sex in interest	-.13	.42

**NOTE**: BDI = Beck Depression Inventory. N = 178 adolescents.

^a ^Principal component analysis. Rotation method: Promax with Kaiser normalization.

^b ^= Loadings > 0.50.

Among the models although the 6-factor model had the maximum variance explained (53.9%), when we looked at the the most parsimonious models it was the 3-factor (36.9%) and 2-factor models (30.5%). As the variance explained between the 3-factor and 2-factor models were not significntly different and when we applied the Louis Thurstone's interpretability criteria for the different models, the 3-factor and 2- factor models had the closest fit with the interpretability criteria, we chose to explain our factor structure with the 2-factor model.

## Discussion

We felt that a measure specifically validated to screen for depressive symptoms in adolescents in India was required. This study was the first one to evaluate the psychometric properties of BDI in India among adolescents and demonstrates the validity as well as diagnostic accuracy of BDI as a screening measure among adolescents attending school while used by paediatricians. These findings build upon previously published validation data, which has demonstrated the use of BDI in many setting and culture [12,13,20-22].

The diagnostic accuracy parameters of sensitivity and specificity were achieved for screening and diagnostic purpose in our study. For the screening procedures a threshold score of ≤ 5 yielded the maximum clinical efficiency with a sensitivity and specificity of 90.9% and 17.6% respectively. Where as for a diagnostic use a threshold score of ≥ 22 provided a sensitivity and specificity of 27.3% and 90.0% respectively, which are comparable with previous study among adults [23] and adolescents [24] in primary-care settings. Like the past studies, we also have recommended two cut-off scores instead of score ranges to classify the severity of depression as was originally used [11,12] as we have validated BDI as screening or diagnostic measure of depressive syndromes,

Among the different parameters used to assess the reproducibility, the inter-rater reliability is not appropriate for the BDI as it is a self-rated measure [12] and therefore only test-retest reliability was done in this study. The test-retest reliability was found to be good and is comparable with that of the test-retest reliability of 0.48 to 0.86 reported at 2 to 6 weeks [25].

The face and content validity of BDI as a measure for depression has long been established by consensus among clinicians [12] and it has been shown that the BDI items are consistent with six of the nine Diagnostic and Statistical Manual, Edition III (DSM-III) categories of symptom clusters of depression [25]. The content validity of BDI in this study was as good as reported elsewhere [26].

The method of Cronbach's alpha was applied to evaluate the scale item homogeneity. The internal consistency of BDI in our study was high and in agreement with what has been reported in other studies. Among the previous studies, the internal consistency for the BDI has ranged from .73 to .92 with a mean of .86 [10,25].

The convergent validity of the BDI has not been documented in the adolescent population with other psychometrically sound instruments for depression. However, the convergent validity of BDI with Hamilton Psychiatric Rating Scale for Depression has been (0.73), Zung Self Reported Depression Scale (0.76) and the MMPI Depression Scale (0.76) among adults [25]. In the present study the convergent validity between the BDI and the Children's Depression Rating Scale had been high among the adolescents. The BDI mean score was relatively high with where as the prevalence of clinically diagnosed depression was low and this could have happened because it is known that high BDI scores in the absence of clinical depression can occur when there are non-depressive symptoms like anxiety symptoms [26]. Depressive symptoms among our adolescents could also have occurred because of the developmental stage related environmental issues shaming, self-verification, self efficacy, attachment insecurity, maladaptive coping and attribution styles or environmental factors like parenting issues [27].

The discriminant validity of the BDI in our study was high demonstrating that it can differentiate other psychiatric disorders like Post-traumatic Stress Disorder that can have affective symptoms. On the other hand, as the co-morbidity overlap of PTSD and depression is common, using IES as the discriminant measure could have compromised the discriminant validity from having even higher values. Many past studies have found that the BDI discriminates depressive symptoms from depressive disorder, dysthymic disorders, loneliness, stress and non-psychiatric patients among adults [25,28-32]. Recently, the ability of the BDI to discriminate adolescents with depression from those who are not depressed has also been established [21].

This study demonstrated a two-factor model for BDI. Previous data on the construct validity of BDI has documented two to seven factors, depending on the method of factor extraction [10]. Both the factors, *Factor 1 (Somatic symptoms) and Factor 2 (Mood-negative cognitions) had only 4 and 3 factors *falling under them respectively and in the previous studies also it had been proposed that only a few factors and items are stable [27]. This lack of stability of the construct over studies has been speculated because of the measurement of state and trait of depression by the BDI [25].

A few limitations of this study must be acknowledged. Firstly, the low prevalence of depression in the sample could have limited the power and stability of the sensitivity analyses. Further, recruiting school children with ablility to read and write English atleast at sixth grade level could have introduced some selection bias. Other shortcomings of the BDI are its controversial factorial validity, and poor discriminant validity against anxiety. Lack of a representative norm could have componded the factor structure further and not including adolescents with anxiety symptoms did not address the discriminant validity against anxiety disorders. It should be noted that BDI provides a measure of severity of depressive symptoms and further clinical assessment may be needed for confirmation of a syndrome of depression, thus BDI is not a diagnostic tool for depressive disorders. We have used the term 'diagnostic accuracy' only to be in concordance with the STARD guidelines for reporting validation studies.

## Conclusion

In conclusion, despite these limitations, this study has demonstrated that the BDI has sound psychometric properties in a primary care setting among adolescents while being used by paediatricians. Our study further supports the Beck Depression Inventory as a viable and reliable measure for identifying probable cases of Depressive disorders among adolescents.

## Abbreviations

AUC = area under the curve

BDI = Beck Depression Inventory

CDRS-R = Children's Depression Rating Scale-Revised

DSM-III = Diagnostic and Statistical Manual, Edition III

ICD 10 = International Classification of Diseases (Clinical Guidelines Diagnostic Criteria version) 10^th ^edition

IES-8 = Impact of Events scale – 8 item version

ROC = Receiver operating characteristic

Sn = Sensitivity

Sp = Specificity

## Competing interests

The author(s) declare that they have no competing interests.

## Authors' contributions

MB was involved in conception, drafting and revising the final draft. PDM and SR were involved in conception and approving the final version of the manuscript. PSSR was involved in conception, designing, data analysis and interpretation, drafting and approving the final version. All authors read and approved the final manuscript.
